# Accessing the Influence of Consumer Participation on Purchase Intention Toward Community Group Buying Platform

**DOI:** 10.3389/fpsyg.2022.887959

**Published:** 2022-06-28

**Authors:** Tanaporn Hongsuchon, Jing Li

**Affiliations:** ^1^Chulalongkorn Business School, Chulalongkorn University, Bangkok, Thailand; ^2^Intellectual Property Research Institute, Xiamen University, Xiamen, China

**Keywords:** community group buying, consumer participation, community identity, privacy concern, purchase intention

## Abstract

The rapid development of community group buying platforms has attracted a huge attention from both the practical and academic communities. Although previous research has explored the influence patterns of community group buying platform on the customers’ purchase intention, there are limited studies on how customers’ purchase intention is influenced by their participation behavior. Therefore, based on social identity theory, this study constructs a theoretical model of consumer participation influencing users’ purchase intention through community identity in the community group purchase context, and examines the moderating role of users’ privacy concerns in this process in conjunction with privacy concern theory to systematically explore the role of consumer participation on purchase intention and its boundary conditions. In this study, the data collected from 532 valid samples are analyzed by structural equation modeling. The results of the study found that customer engagement behavior had a significant effect on purchase intention through the mediation of community identity, where privacy concerns negatively moderated the effect of community identity on purchase intention. The study reveals the intrinsic mechanism of customer engagement influencing purchase intention and its boundary conditions, which provides the suggestions for the marketing management and business practice of community group platforms.

## Introduction

Since 2020, there have been some major changes in community group buying platforms in China, with the launch of new brands/platforms, and a lot of budget being invested in such platforms (Li et al., 2020; Hu and Shu, 2021). Obviously, with the rapid development of social media and the popularization of community group buying APP (Yang et al., 2021), in-depth analysis of customer engagement to establish a good relationship between consumers and community group buying platforms is extremely important for community group buying platforms in a competitive business environment to maintain consumers’ continuous use. Consumer participation to a certain extent causes the occurrence of psychological changes in consumers, which leads to their purchase intentions and behaviors. It profoundly affects the behavior, interpersonal interaction rules, and related performance of members within the community group buying platform, and has become a frontier topic of current research in the field of virtual environments.

It is now believed that consumers are likely to be influenced by their engagement behaviors to purchase the community group buying platform itself and the products or services it offers, and that consumer usage behaviors are the most essential element in maintaining the “consumer-community group buying platform” relationship. The consumer behavior is the most essential element of the relationship between the “consumer and the community group buying platform,” and is the basis for the success of the community group buying platform (Hu and Shu, 2021). Community group buying platform consumer “participation behavior – willingness to buy” problem research is crucial, become community group buying platform business model innovation, marketing management changes, and theoretical research boom (Li et al., 2020). The research boom is mainly driven by two forces: the practical needs of community group buying platforms and the theoretical needs of academics (Hsu et al., 2018). Both hope to assist community group buying platforms to explore a new way of operation and development as a way to gain competitive advantage (Yan and Li, 2021). Although the above inferences can be drawn from both theoretical and practical viewpoints, and a series of valuable results have been achieved in the study of customer engagement on purchase intention. However, the relationship between consumer participation and community group buying platform consumer behavior has been studied in a wide range of areas, and deeper theoretical logic, and the ways in which community group buying platform consumer involvement works still need to be further explored.

Numerous studies have also found that, for example, online interactions between consumers can enhance customer engagement and improve perceptions of the integrity and goodwill of the community group buying platform; and the use of avatars can rely on customer engagement to enhance the sense of identity and strengthen the consumers’ perceptions of the community group buying platform (Deng, 2016; Hsu et al., 2018; Kamboj et al., 2018). However, previous literature has mostly focused on individual consumer factors and system characteristics, while the mediating effect of community identity as a driver of consumer use of IT products or services is lacking, and less attention has been paid to the impact of community identity on the path between user customer engagement and purchase behavior (Li, 2019). In order to expand and enrich the existing research field, it is necessary to explore how consumer participation affects consumer purchase intentions in a virtual context.

Based on the above discussion, this study aims to construct a moderated mediating model around the relationship between consumer participation and purchase intention in the context of community group purchase usage by combing the research literature on consumer participation theory, community identity, and privacy concerns to explore the mediating psychological mechanisms and boundary conditions by which consumer participation affects purchase intention. Specifically, this study employs structural equation modeling (SEM) to delve into its inherent theoretical logic and influence mechanisms to address and answer the following questions. First, this study evaluates the effect of community identity on driving purchase intention in the context of community group purchase usage. Second, this study estimates the effect of different components of customer engagement (information sharing, responsiveness taking, and interpersonal interaction) on community identity in the context of community group purchasing. Third, this study discusses the role of community identity in mediating the relationship between consumer participation and purchase intention in the context of community group purchasing. Fourth, this study assesses the moderating role of customers’ privacy concerns in the relationship between their community identity and purchase intention in the context of community group purchasing.

## Theoretical Foundation and Hypotheses Development

### Social Identity Theory

The concept of community identity is a further application and development of social identity in specific contexts (Sun et al., 2021). Tajfel (1982) defined social identity as “an individual’s perception of belonging to a particular social group and that group membership has emotional and value significance for him or her.” Since then, scholars have mostly followed this definition (Li et al., 2017). Tajfel and Turner (1986) found that individuals in a particular social group setting are characterized by a tendency to embody or express self-esteem. In other words, individuals possess a tendency to seek self-concept. In a group participation setting, interactions between groups can influence individual emotions, such as positive values. The values espoused by a group tend to reflect the identity of individuals within that group (Li and Gao, 2019). Individual identity is a prerequisite for the establishment of group identity, and the individual’s evaluation of the group is based on the judgment of the characteristics and value propositions of other groups (Ashforth and Mael, 1989). The individual’s evaluation and judgment make the group’s self-concept to be strengthened, thus constantly enhancing the individual’s perception of its superiority (Algesheimer et al., 2005; Muniz and Schau, 2005; Bagozzi and Dholakia, 2006). Therefore, when the individual needs to get the superiority of the group, the individual will choose to join the group with which he or she has commonalities. In this particular group, the individual receives a sense of superiority due to membership, such as superior status, superior position, and superior image. Community identity is a special form of expression of social identity in which people participate in interaction, communication, and shopping by creating a virtual identity for themselves, which plays a key role in interpersonal communication in a technologically mediated environment (Turkle, 1995). It reflects the virtual self-role that the user wants to express and present to others. On the community group buying platform, users can build and express themselves using freely scalable symbolic tools (e.g., short videos, personalized signatures, user avatars) (Schau and Gilly, 2003), creating one or even multiple online “virtual identities” to present multiple aspects of themselves. As in the real world, there is a dual motivation for users to participate in the community group buying process to satisfy a sense of belonging and to preserve individuality (Schau et al., 2009), i.e., the construction of a virtual identity reflects the need for community identity in the real world: self-identity through the search for differences between “I” and “we” and community identity through the search for differences between “we” and “they” (Tajfel and Turner, 1986).

Community identity is one of the components of personal self-identity, reflecting the connection of the self to a specific virtual environment based on direct or indirect experience, and a spiritual dimension. This concept can be used to explain the individual’s relationship with the physical environment as a synthesis of affective and symbolic connections to a specific physical environment that determines who the user is and also contains the emotional meaning formed by causal expressions and confirmations (Tajfel, 1982). The results of Algesheimer et al. (2005), using the virtual society as the subject of the study and analyzed by SEM, indicate that consumers’ identification with the brand community has a significant positive impact on their brand purchasing behavior. Bagozzi and Dholakia (2006) suggest that community affiliation promotes brand purchasing behavior among community members in virtual communities, and Zheng et al. (2014) show that community identification can indirectly influence consumers’ brand-related purchasing behavior through brand identification. Yu (2018) states that customers’ brand community identity can influence their attitudes and behaviors toward their brands. Hu and He (2020) further validated the findings of Yu (2018) through an empirical study that brand community customer identification has a significant positive impact on their purchase intentions and behaviors. Obviously, in terms of research on the relationship between community identity and customer buying behavior. Community group buying platform is not only a platform for communication between community group buying platform and consumers, but also has gradually become an important channel for the sales of community group buying platform related products. Therefore, it is necessary to evaluate the influence mechanism of customers’ purchase intention in the context of community group buying platform usage in order for community group buying platform to grasp the market trend and maintain its competitive advantage. However, the role of community group buying platform identity on purchase intention has been studied by scholars, but this part of the research is mostly qualitative analysis rather than quantitative empirical evidence. Based on the above analysis, this study extends the applicability of community identity to this new research area, explores the influence mechanism of consumer participation on customers’ purchase intention, and focuses on the mediating role of community identity in it.

### Consumer Participation

Kelley et al. (1990) argued that consumer participation connotes the behavior of information sharing between individuals in a stimulating environment. According to Belén del Río et al. (2001), consumer participation is a behavioral concept that refers to the resources provided and the behaviors given by customers in the process of service production or delivery, and consumer participation is a process-oriented behavioral concept (Wang, 2022). Based on this, Groth (2005) refined the relevant research of the aforementioned scholars and pointed out that “customer citizenship behavior is the behavior that customers give in the process of service and delivery, and divided customer citizenship behavior into two categories: in-role behavior refers to customer cooperative production behavior, while out-role behavior refers to other customer participation behaviors involved in the process of service and delivery” (Zhang et al., 2021). With further research and expansion on consumer participation, consumer participation has been considered as a multidimensional construct (Bettencourt, 1997; Ennew and Binks, 1999; Claycomb et al., 2001; Lloyd, 2003). Lloyd (2003) pointed out through empirical analysis that “consumer participation is made up of three dimensions: information sharing, responsible behavior, and interpersonal interaction.”

A further study, Yang and Chen (2017), in which online brand communities were the subject, classified customer engagement into information sharing, responsibility taking, and interpersonal interaction based on the synthesis of previous studies. Therefore, this study concludes that customer engagement in the context of community group purchasing is “the result of a combination of both psychological and behavioral aspects of customer engagement, which refers to the resources and behaviors that customers put into the process of service production and delivery to accomplish value creation” (Claycomb et al., 2001; Groth, 2005; Wang, 2019). Consumer participation consists of three main dimensions, namely information sharing, responsibility taking, and interpersonal interaction. Information sharing in the community group buying platform refers to information sharing between community members and community managers to facilitate community services to meet the needs of community members, as well as information sharing between community members regarding the purchase, use, and experience of products/services.

In general, information sharing among community members is the main part of information sharing in the community group buying platform (Li et al., 2016). Responsibility taking refers to the clarification of the responsibilities of community members and community managers. For customer participation, it is more about community members cooperating with and assisting community managers to make the delivery of community services smoother, and at this time, customers have become a new platform for community group buying platform to collect information about product innovation. Since the community group buying platform is more concerned about whether customer participation can bring new ideas and inspiration to the community group buying platform, it often collects information on the development and promotion of new products in the virtual button brand community (Bartl et al., 2012; Wang, 2019; Zheng et al., 2022). Interpersonal interaction in community group buying platform consists of two main aspects: one is the interaction between community members and community managers, and the other is the interaction between community members and each other. Moreover, interpersonal interaction in the community group buying environment helps to promote community services to meet the needs of community members (Wang, 2019). The interpersonal interaction among community members helps to build a good community atmosphere and promote mutual trust, support, and cooperation among community members, which constitute the main aspects of interpersonal interaction in the community group buying platform.

### Hypothesis Development

As a typical virtual community, users in the community group buying platform use scenario to join the community based on the demand of seeking self-worth or due to the recommendation of others, and the community identity can bring actual benefits and values to customers. The community identity can bring actual benefits and values to customers, and customers can obtain corresponding values through the community group buying platform, thus creating community identity (Wang, 2019). The study concluded that consumer participation in the production of community group buying platform can reduce the manpower input of community group buying platform, which not only allows community group buying platform to allocate human resources rationally and improve productivity, but also can utilize the wisdom of customers to improve the service quality in a more targeted manner and meet the relevant needs of consumers (Li et al., 2016). Aaker (1997) pointed out that community group buying platform must continuously attract customers to build brand identity to form brand equity, and brand identity comes from the process of consumer participation in the production and dissemination of community group buying platform products/services. In the scenario of community group buying platform, consumer participation helps the formation of community identity (i.e., the formation of identification with that community group buying platform) and, generating a sense of psychological gain.

With the improved standard of living in modern society, consumers spend most of their time in a state of emotional anxiety to cope with the uncertainty in their lives (Yun et al., 2019). When customers generate a certain consumer behavior, this is likely to make them more anxious when there is asymmetric information about the product/service. An effective solution to this problem is the consumer participation community group buying platform, where consumers can choose to participate in a virtual brand community and use the information communication in this virtual platform to effectively combat the information asymmetry and the associated risk of uncertainty in the shopping process; thus the virtual brand community can be used to counteract the information asymmetry and the risk of uncertainty in the shopping process, thus alleviating customers’ choice phobia and anxiety and making them happy. Consumer participation in community group buying platforms improves individual knowledge and cognitive skills, thanks to the space that community group buying platforms provide for individuals to share, exchange, and transfer information. Individuals will get feedback from others when they provide information about products/services to their customers, and in this process, the knowledge and ability of customers will be exchanged and improved together. In addition, the community group buying platform provides an online platform for individuals to exchange, communicate, and discuss with each other. Therefore, in the community group buying platform scenario, individuals can get more opportunities to communicate with others and thus gain more pleasure, and these emotional benefits will enhance customers’ identification with the platform. Based on the aforementioned arguments, this study hypothesize the following:

H1a: Information sharing is positively associated with community identity.

H1b: Responsibility taking is positively associated with community identity.

H1c: Interpersonal interaction is positively associated with community identity.

Social identity theory suggests that identity works by two main mechanisms: on the one hand, there is the effect on the individual’s self-esteem, and on the other hand, the distinction the individual makes between inside and outside the group (Tajfel, 1982). Firstly, individuals identify themselves with a group through self-categorization and develop a sense of identity with that group. Individuals increase their self-esteem through this positive social identity (Turner, 1985). Secondly, social identity allows individuals to develop in-group identity and out-group bias. In-group identification enhances individuals’ self-esteem, while out-group prejudice leads individuals to in-group advocacy and out-group exclusion behaviors. With the increase and enrichment of the literature on community identity, academics have begun to verify the applicability of social identity theory in the application of virtual communities because the concept of community group buying platform’s customer community identity, from its introduction to its continuous development, is essentially a social identity (Wang et al., 2013). Since community group buying platform community identity has the same internal logic as social identity in essence, the past studies of scholars are summarized and analyzed to deeply explore the meaning and connotation of community group buying platform identity. It can be seen that in the community group buying platform scenario, customer community identity is a special kind of social identity in its essence, and therefore has the fundamental properties and functions of social identity.

According to the theory of community identity, customers divide themselves into a community group buying platform, agree and accept the system and norms of the community group buying platform, and participate in the activities of the community group buying platform. In this process, customers become dependent on and identify with the community group buying platform, and this psychological change in the process stimulates the generation of community identity. Therefore, customers see the community group buying platform as a key element of self-presentation and identity, and by enhancing the connection between themselves and the community group buying platform, they increase their perception of the value symbolic ability of the community group platform, which in turn has a significant positive impact on their intention to continue using the community group buying platform. Thus, community identity plays an important role in the construction of the community group buying platform and continues to influence customers’ behavior related to participation in the community as they shop through the platform. Studies in the context of virtual community platforms have also found that individuals’ community identity influences their intention to engage in related behaviors (Algesheimer et al., 2005).

Bagozzi and Dholakia (2006) further found that community identity in virtual situations not only has a positive effect on purchase intentions, but also indirectly influences individuals’ behavioral intentions through the mediating role of brand identity. In addition, Schau et al. (2009), based on an empirical study, pointed out that individual community identity in virtual platforms stimulates consumers’ brand perceptions and brand behaviors, and this view was confirmed by He and Yan (2018). He and Yan (2018) study concluded that community identity has a significant effect on their loyalty behavior. It can be seen that customers’ community identity in the virtual environment will further stimulate purchase intention and other related behaviors. As such, this study proposed the following hypothesis.

H2: Community identity is positively associated with purchase intention.

The above analysis shows that in community group purchase usage scenarios, consumer participation has a positive impact on their community identity (Aaker, 1997; Carlson et al., 2008; Li et al., 2016), and customer community identity can in turn have a positive impact on purchase intention (Belén del Río et al., 2001; Teo et al., 2003; Algesheimer et al., 2005; Bagozzi and Dholakia, 2006; Schau et al., 2009; He and Yan, 2018). It can be seen that community identity is the mediating variable that connects consumer participation and purchase intention in the context of community group buying platform usage.

Further research suggests that community identity is driven by consumer participation, which in turn motivates customers to generate a series of related behaviors, such as sharing shopping behavior, recommending products, and willingness to pay (Lin et al., 2017). It can be seen that community identity plays a connecting role between consumer participation behavior and purchase intention. Based on the above analysis, this study finds that in the context of community group buying platform use, customer community identity has a connecting role between consumer participation and purchase intention, i.e., community identity plays a role in the relationship between consumer participation pairs and purchase intention. In other words, community identity plays an intermediary role in the relationship between consumer participation and purchase intention. Based on a comprehensive analysis of the above, this article thus hypothesizes:

H3a: Community identity plays a mediating role between users’ information sharing and purchase intention.

H3b: Community identity plays a mediating role between users’ responsibility taking and purchase intention.

H3c: Community identity plays a mediating role between users’ interpersonal interaction and purchase intention.

In virtual environments (e.g., social media or virtual platforms), privacy concerns refer to users’ perceptions and concerns about the collection, acquisition, monitoring, and use of personal information (Jia et al., 2021). With the widespread use of the Internet and big data technologies, lots of social media platforms are recognizing the value and importance of consumer data. Collecting, storing, and using consumers’ private data in various direct or indirect ways is gradually becoming a regular marketing practice for social media platforms (Krafft et al., 2017; Yu, 2018). For example, the ability of companies to hold and analyze massive amounts of information, including consumers’ personal privacy data, is the basis for enabling behavioral targeting and product recommendations. At the same time, due to the relative lag of consumer privacy-related legislation and industry regulations in China, public policies have not yet been able to form effective legal constraints and institutional regulations on enterprises, and the importance that enterprises attach to obtaining customer privacy data and the neglect of protecting it coexist. Incidents of large-scale leakage of customer privacy are frequent and even escalate into scandals that seriously damage consumers’ interests and corporate reputation (Janakiraman et al., 2018). And with the increasing public awareness of personal privacy, consumers’ concern about companies’ behavior in collecting, keeping and using personal privacy data is increasing. Moreover, they also show a tendency to give substantial feedback on these actions of companies.

Consumers not only allow companies to collect information about themselves for benefits such as convenience or experience (Gabisch and Milne, 2014), but also create concerns about privacy leaks or misuse of information. The privacy concern in the consumer domain has become an inevitable and important issue in the virtual environment (Ashforth and Mael, 1989; Martin and Murphy, 2017). It has been shown that “in general, individuals psychologically perceive that they can share and communicate carefree on a secure virtual platform because they perceive high levels of perceived value and develop a sense of belonging in their continued use of that virtual platform. The impact of the privacy breach results is that users with higher privacy concerns are concerned about the collection, control, and use of personal information during the use of the medium, thus creating a perceived loss factor for using that community group purchase” (Wang et al., 2021). This illustrates that if a user’s privacy concerns are more sensitive, then that user will expect enhanced privacy protection in their purchase intentions, resulting in lower purchase intentions. It can be seen that in the context of community group purchase usage, the influence of community identity on purchase intention is not significant for users with low levels of privacy concerns. Users with high levels of privacy concerns, on the other hand, still had an effect on purchase intention, but did not have the same lack of privacy protection and security awareness as users with low levels of privacy concerns. Therefore, this study infers that the relationship between community identity and purchase intention is influenced by privacy concerns. Thus, this paper derived the following hypothesis:

H4: Users’ privacy concern plays a moderating role between community identity and purchase intention.

**FIGURE 1:**
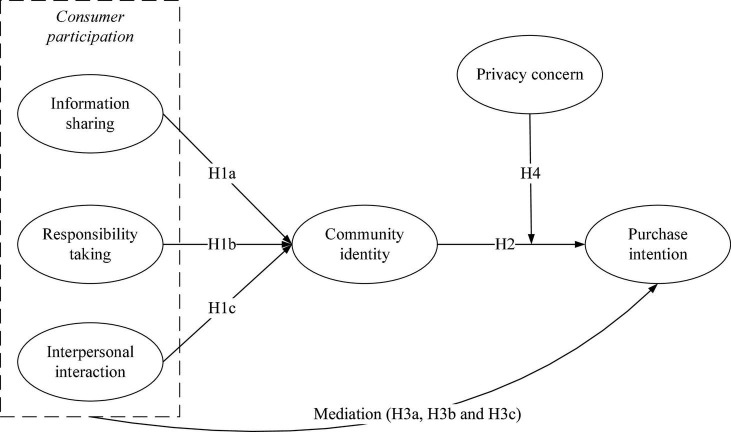
Theoretical model.

In summary, this study proposes a research model as shown in Figure 1.

## Materials and Methods

### Participants and Procedure

The data collection of this study fully considered the representativeness of the sample, and selected “Chengxin Youxuan,” “Duoduo Maicai,” and “Meituan Youxuan” users. This is because “Meituan Youxuan” belongs to a new generation of community group buying characterized by platform, “Chengxin Youxuan” belongs to a comprehensive community group buying integrating graphic information, video advertisement, and chat, and “Duoduo Maicai” represents a traditional community group buying platform. All three have a sizeable user base in China. In this study, 532 valid samples were collected from 12 July 2021 to 15 October 2021. The results of descriptive statistics are shown in Table 1.

**TABLE 1:** Descriptive statistical analysis.

Variables	Item	Frequency	%	Cumulative %
Gender	Male	232	43.61	43.60
	Female	300	56.39	100
Age (year)	20 years old or less	60	11.27	11.27
	21∼30 years old	182	34.21	45.48
	31∼40 years old	176	33.08	78.56
	41∼50 years old	86	16.16	94.72
	51 years old or above	30	5.28	100
Marriage	Married	214	40.23	40.23
	Unmarried	318	59.77	100
Profession	Civil servants	140	26.31	26.31
	Employees of enterprises	200	37.59	63.90
	Retirees	128	24.06	87.96
	Other	64	12.04	100
Education	College and blow	172	32.33	32.33
	Undergraduate	190	35.71	68.04
	Master’s degree and above	170	31.96	100
Consumption (RMB)	Below 2,000 RMB	120	22.55	22.55
	2,000∼3,999 RMB	162	30.45	53.00
	4,000∼5,999 RMB	160	30.08	83.08
	6,000 RMB or more	90	16.92	100
Continuous use time (year)	Within 6 months	182	34.21	34.21
	Less than 1 year	148	27.81	62.02
	1∼2 years	130	24.43	86.45
	Over 3 years	72	13.55	100

### Measures

Consumer participation, a 12-item scale, proposed by Ennew and Binks (1999), Claycomb et al. (2001), Groth (2005), and Kim et al. (2010), were adopted to measure three dimensions of consumer participation, among which 4 items were used to measure information sharing, four items for measuring responsibility taking, and four items for assessing interpersonal interaction. Community Identity scale of this research mainly refers to the research views of Zheng et al. (2017), mainly used to measure consumers’ attitudinal preferences and emotional responses to community group purchasing, including three items. Privacy concern is modified from Son and Kim (2008), Shin (2010), and Tan et al. (2012). Purchase intention, including four items, adopted from Grewal et al. (1998) and Papagiannidis et al. (2014).

All the measurement items for this research were translated from English into Chinese following the back-translation procedure advocated by Cha et al. (2007), and we modified the measurement items according to the actual usage situation of community group buying. These measurement items are rated on a 7-point scale ranging from 1 (strongly disagree) to 7 (strongly agree).

## Data Analysis and Results

### Confirmatory Factor Analysis

The results of the confirmatory factor analysis (CFA) are shown in Table 2. This study evaluates and revises the measurement model of CFA according to the approach of Anderson and Gerbing (1988) *via* IBM SPSS AMOS 24. That is, CFA should demonstrate standardized factor loading, reliability (i.e., Cronbach’s Alpha and composite reliability), and Average Variance Extracted (AVE) for all constructs, and only after these metrics pass the tests, structural model evaluation can be performed (Kline, 2011). Fornell and Lacker (1981) and Hair et al. (2017) clearly stated that when the factor loadings should be greater than 0.7, Cronbach’s Alpha and composite reliability should be greater than 0.7, and AVEs are greater than 0.5, then the measurement model has the convergent validity (as shown in Table 2).

**TABLE 2:** Confirmatory factor analysis.

Construct	Item	Factor loading	Cronbach’s alpha	Composite reliability	Average variance extracted
Information sharing (IS)	IS1	0.708	0.852	0.855	0.598
	IS2	0.722			
	IS3	0.823			
	IS4	0.832			
Responsibility taking (RT)	RT1	0.822	0.895	0.897	0.686
	RT2	0.867			
	RT3	0.856			
	RT4	0.764			
Interpersonal interaction (II)	II1	0.777	0.855	0.857	0.599
	II2	0.750			
	II3	0.750			
	II4	0.817			
Community Identity (CI)	CI1	0.800	0.836	0.838	0.634
	CI2	0.801			
	CI3	0.787			
Privacy concern (PC)	PC1	0.819	0.908	0.908	0.713
	PC2	0.817			
	PC3	0.874			
	PC4	0.865			
Purchase intention (PI)	PI1	0.817	0.909	0.914	0.728
	PI2	0.917			
	PI3	0.941			
	PI4	0.720			

Table 3 reports the discriminant validity for the measurement model. The discriminant validity is an analysis used to test whether any two variables in the theoretical model are identical to each other. If there is no identity between any two variables, then the measurement model has reasonable discriminant validity. This study uses the generic discriminant validity analysis method, namely confidence interval method (Torkzadeh et al., 2003). The confidence interval method is used to confirm the confidence interval of the correlation coefficient between variables. If the confidence interval of correlations between constructs includes zero, the empirical data can’t pass the test of discriminant validity. As Hancock and Nevitt (1999) suggested, bootstrap method was conducted in this research, 95% confidence interval of the correlation coefficient does not involve 1, which shows the discriminant validity among constructs (as shown in Table 3).

**TABLE 3:** Discriminant validity for the measurement model.

Parameter	Correlation	Bias-corrected 95%		Percentile 95%	
		Lower	Upper	Lower	Upper
IS<–>RT	0.735	0.638	0.802	0.643	0.807
IS<–>II	0.662	0.562	0.765	0.548	0.756
IS<–>CI	0.673	0.578	0.761	0.575	0.757
IS<–>PI	0.528	0.418	0.645	0.409	0.631
IS<–>PC	0.455	0.338	0.570	0.334	0.566
RT<–>II	0.672	0.573	0.772	0.570	0.769
RT<–>CI	0.639	0.550	0.717	0.550	0.717
RT<–>PI	0.490	0.371	0.596	0.365	0.590
RT<–>PC	0.424	0.308	0.532	0.310	0.534
II<–>CI	0.640	0.529	0.730	0.525	0.726
II<–>PI	0.602	0.484	0.698	0.479	0.697
II<–>PC	0.492	0.356	0.607	0.359	0.607
CI<–>PI	0.627	0.516	0.742	0.500	0.731
CI<–>PC	0.596	0.465	0.714	0.464	0.712
PI<–>PC	0.426	0.293	0.547	0.289	0.542

*IS, information sharing; RT, responsibility taking; II, interpersonal interaction; CI, community identity; PI, purchase intention.*

### Structural Model Analysis

The results of the model fit degree are shown in Table 4. The study by Jackson et al. (2009) states that structural models should report model fit metrics as a way to assess, correct, and judge the goodness of measurement models. As suggested by Jackson et al. (2009), Normed Chi-square (χ^2^/df), SRMR, TLI (NNFI), and CFI are the common metrics used to test the fit of research models. Therefore, academics usually use these nine metrics to test whether the model fit is good or not. In principle, the lower χ^2^ is better, but since χ^2^ is very sensitive to the sample size, so it is evaluated with the assistance of χ^2^/df, whose ideal value should be less than 5. The judgment criteria of all indicators are shown in Table 4. The results of model fitness are shown in Table 4, and all of them meet the suggested criteria of Jackson et al. (2009). Therefore, the structural model has a good fit in this study.

**TABLE 4:** Model fit criteria and the test results.

Model fit	Criteria	Model fit of research model
Normed Chi-square (χ^2^/df)	<5	4.433
SRMR	<0.08	0.058
TLI (NNFI)	>0.9	0.901
CFI	>0.9	0.916

The path coefficients are shown in Table 5. Information sharing (IS) (*b* = 0.354, *p* < 0.001), responsibility taking (RT) (*b* = 0.130, *p* < 0.05) and interpersonal interaction (II) (*b* = 0.296, *p* < 0.001), are positively associated with community identity (CI). Therefore, H1a, H1b, and H1c are established. Community identity (CI) (*b* = 0.832, *p* < 0.001) is positively associated with purchase intention (PI). Therefore, H2 is established.

**TABLE 5:** Regression coefficient.

	Unstd. coefficient	S.E.	*z*-value	Std. coefficient	*p*
H1a	0.354	0.067	5.289	0.362	***
H1b	0.130	0.051	2.576	0.167	*
H1c	0.296	0.053	5.628	0.341	***
H2	0.832	0.061	13.588	0.692	***

*p-value < 0.05; ***p-value < 0.001.

The results of the mediation effect analysis are shown in Table 6. In this study, SEM was used to analyze the mediation effect, and the standard error of the mediation effect was first estimated using bootstrap estimation technique, and then the significant level of the mediation effect was further calculated. According to Hayes (2009), a mediation effect is indicated if “0” does not include the 95% confidence interval of bias-corrected, the *z*-value is greater than 1.96, and the *p*-value is less than 0.05.

**TABLE 6:** The analysis of mediation effect.

Effect	Point estimate	Bootstrap 1000 times			
		Bias-corrected 95%		Percentile 95%	
		Lower bound	Upper bound	Lower bound	Upper bound
Total effect: IS→PI	0.244	0.040	0.472	0.017	0.465
Indirect effect: IS→CI→PI	0.185	0.087	0.361	0.078	0.330
Direct effect: IS→PI	0.058	–0.169	0.298	–0.170	0.292
Total effect: RT→PI	0.044	–0.135	0.208	–0.128	0.215
Indirect effect: RT→CI→PI	0.078	0.005	0.170	0.001	0.166
Direct effect: RT→PI	–0.034	–0.203	0.113	–0.184	0.120
Total effect: II→PI	0.454	0.254	0.671	0.249	0.660
Indirect effect: II→CI→PI	0.132	0.044	0.272	0.043	0.268
Direct effect: II→PI	0.323	0.088	0.544	0.089	0.544

*IS, information sharing; RT, responsibility taking; II, interpersonal interaction; CI, community identity; PI, purchase intention.*

Specifically, the total effect of information sharing on purchase intention is 0.244. At the 95% confidence level, “0” does not include the bias-corrected 95% confidence interval range, the *z*-value > 1.96, and the *p*-value < 0.05. Therefore, a total effect exists. The indirect effect is 0.185, “0” does not include the bias-corrected 95% confidence interval range, the *z*-value > 1.96, and the *p*-value < 0.05. Therefore, there is an indirect effect. The direct effect is 0.058, “0” does not include the Bias-corrected 95% confidence interval range, the *Z*-value > 1.96, and the *p*-value < 0.05. Therefore, a direct effect exists.

The indirect effect of responsibility taking on purchase intention is 0.078, “0” does not include the bias-corrected 95% confidence interval range, the *z*-value > 1.96, and the *p*-value < 0.05. Therefore, there is an indirect effect. The indirect effect of interpersonal interaction on purchase intention is 0.132, “0” does not include the bias-corrected 95% confidence interval range, the *z*-value > 1.96, and the *p*-value < 0.05. Therefore, there is an indirect effect. In the same analytical approach, the results of the study show that H3a, H3b, and H3c are supported.

The moderation effect is reported in Table 7. In the present study, privacy concern (PC) is the moderating variable. The results of SEM have shown that the moderation effect of community identity (CI) × privacy concern (PC) on purchase intention (PI) is −0.051 (*z* = | −2.010| > 1.96, *p* < 0.05), implying the presence of a positive moderating effect of privacy concern (PC) on the relationship between community identity (CI) and purchase intention (PI). Specifically, the slope of community identity (CI) on purchase intention (PI) increases negatively by −0.051 units for each 1-unit increase in the moderating variable privacy concern (PC). That is, community identity (CI) has a negative moderating effect. Therefore, H4 is verified.

**TABLE 7:** The analysis of moderation effect.

DV	IV	Estimate	S.E.	*Z*-value	*p*
Purchase intention	Community identity	0.726	0.063	11.536	***
	Privacy concern	0.129	0.046	2.797	**
	Community identity × Privacy concern	−0.0051	0.026	−2.010	*

*p-value < 0.05; **p-value < 0.01; ***p-value < 0.001.

## Research Results and Discussion

### Conclusion

First, the effect of community group buying platform consumer participation on community identity is verified. The results of this study indicate that consumer participation has an influential role on community identity, which is consistent with existing research (Yun et al., 2019). In the context of community group buying platform, asymmetric information about products/services is likely to make customers more anxious when they engage in a certain consumer behavior. The effective way to solve this problem is the consumer participation community group buying platform, where consumers can choose to participate in the virtual brand community and use the information communication in the virtual platform to effectively combat the information asymmetry and the associated risk of uncertainty in the shopping process, so as to improve the knowledge and cognitive ability of individuals, which is due to the fact that the community group buying platform provides individuals with a space for information sharing, communication, and transmission. Individuals will get feedback from others when they provide product/service related information to other users, and in this process, the knowledge and ability of customers will be exchanged and improved together. In addition, the virtual group buying platform provides a place for users to be able to communicate. This allows users to have the opportunity to communicate with others and gain more pleasure, and these emotional benefits will enhance the customers’ identification with the platform. The community group buying platform creates an interactive context for customers, providing them with a channel to communicate and share with other customers, thus making them happier and increasing their sense of identity and belonging to the community platform. Therefore, the empirical test results of this study found that consumer participation in community group buying platform can positively affect their community identity. Thus, it is verified that consumer participation in community group buying platform context has a positive effect on customers’ community identity and is a very significant effect on enhancing members’ community identity.

Second, the effect of community group buying platform customers’ community identity on purchase intention was examined. The result indicates that customer community identification has a significant effect on their purchase intention in the context of community group buying platform usage. The findings are consistent with those of Belén del Río et al. (2001), Bagozzi and Dholakia (2006), and He and Yan (2018). Belén del Río et al. (2001) examined the relationship between community identity and purchase intention at both the individual and social identity levels, and found that both individual and social identity have a facilitating effect on customers’ purchase intention, and the effect of individual identity on purchase intention is stronger than that of social identity. Bagozzi and Dholakia (2006) further found that the virtual context. Bagozzi and Dholakia (2006) further found that community identity in virtual situations not only has a positive effect on purchase intentions, but also indirectly influences individuals’ behavioral intentions through the mediating role of brand identity. In addition, Schau et al. (2009), based on an empirical study, pointed out that individual community identity in virtual platforms stimulates consumers’ brand perception and brand behavior, which is confirmed by He and Yan (2018). He and Yan’s (2018) study concluded that community identity has a significant effect on their loyalty behavior. Therefore, this study concludes that community group buying platform context, customers’ community identity stimulates their purchase intention and thus has a significant impact.

Third, the mediating role of community identity between consumer participation and purchase intention is examined. This result shows that community identity mediates the relationship between consumer participation and purchase intention, which is consistent with existing research. Lin et al. (2017) further showed through an empirical study that individual and social identities of customers are influenced by customer participation, which leads to a series of related behaviors, such as sharing shopping behavior, recommending products, and willingness to pay. It can be seen that community identity plays a role in linking customer engagement behavior and purchase intention. Based on the above analysis, this study suggests that community identity plays a mediating role in the relationship between customer participation and purchase intention. To this end, a theoretical model of customer engagement behavior influencing users’ purchase intention through community identity in a local community group purchase context is constructed. This study uses Orange Heart Preferred, Duo Buy, and Meituan Preferred as the samples for data collection and investigation to explore the role of consumer participation on purchase intention and its boundary conditions. The results of the study revealed that community identification is an intermediate variable between consumer participation and customer purchase intention. consumer participation is a mediating effect on the purchase intention and purchase behavior of community residents, and its main means is participation in community activities. By communicating with other consumers in community activities, sharing information, and sharing responsibilities as a member of the community group buying platform, customers form a sense of identification with the community group buying platform, and on this basis, increase their purchase intentions. The results of the empirical study show that the enhancement of customers’ purchase intention by customer participation is predicated on allowing consumers to establish their identification with the community group-buying platform.

Fourth, this study proved the moderating effect of privacy concerns on the relationship between community identity and purchase intention in community group buying platform. The findings are consistent with the logical reasoning of Krafft et al. (2017), Yu (2018), and Wang et al. (2021). It is hypothesized that the main reason for this may be the role of privacy concerns not only in terms of its direct impact, but also in terms of its effectiveness as an important moderating variable that can change the effect of other marketing factors on consumer behavioral responses. Moreover, there is a wide range of contexts in which privacy concerns play a moderating role (Tan et al., 2012; Wang et al., 2021). Specifically, privacy concerns affect the strength of the role of customer community identity on the intention to use community group buying; inhibit consumers with community identity on community group buying from showing positive rather than negative attitudes; inhibit consumers from having significant resistance to community group buying based on personalized recommendation technology, which in turn inhibits purchase intention and changes the effectiveness of consumer community identity on enhancing purchase intention and behavior. Not only that, privacy concern also decreases community group buying users’ distant willingness to disclose privacy, and there is a non-negligible gap between the distant willingness and the behavior itself, which is the root cause of the contradiction between privacy attitudes and behaviors (Hong et al., 2019). From the perspective of moderating focus theory, the difference in the psychological characteristic of privacy concern causes consumers to show negative moderating effects on the positive outcomes of community identification on community group buying.

### Theoretical Contributions

First, this study introduces the theory of consumer participation into the context of community group purchasing, which expands the research context of consumer participation theory. In recent years, the theory of consumer participation has been a hot topic of attention in both theoretical and practical circles, and it has become a research hotspot for further enrichment and development in the subject areas of product innovation, customer curiosity, and customer innovation. However, with the booming and widespread use of community group buying platform, the differences between the community group buying platform context and the virtual environment in the broad sense are more obvious, except for the similarities, such as user characteristics as likes, comments, and retweets. For example, users’ characteristics such as liking, commenting, and retweeting, and the boundary between “virtual-reality” in the context of community group buying platform use are in constant conflict. Based on this, this study applies the theory of consumer participation to the local community group buying platform usage context, extending a new application context for the enrichment of the theory of consumer participation.

Second, a theoretical model of consumer participation influencing purchase intention is constructed. Based on the virtual context perspective, this paper constructs a theoretical model of consumer participation to predict users’ willingness to buy, discuss in depth the relationship between different components of consumer participation, community identity, and willingness to buy, and validates the relationship between community group buying and platform identity through the relevant analysis of identity theory. The relationship between platform identification and purchase intention is verified through the correlation analysis of identity theory. The results show that consumer participation stimulates the generation of their purchase intention mediated by community identity, where privacy concerns negatively moderate the effect of community identity on purchase intention. This study reveals the intrinsic mechanism of customer generation influencing purchase intention and its boundary conditions, which provides a reference for the innovative management and business practice of community group platform.

Third, this paper explores and examines the boundary conditions of privacy concerns in the process of consumer participation influencing purchase intention in the community group purchase usage context. In this study, privacy concerns are introduced as a moderating variable in the community group purchase usage context to extend the existing research on consumer participation influencing purchase intention and the intermediate mechanisms, and to confirm the variability of users’ purchase intention. This study found that users’ awareness of the role of community identity on purchase intention in the community group purchase context is inhibited by privacy concerns in addition to users’ attitudes and behaviors that change their purchase intention, indicating that users’ desire to protect privacy and their privacy control behaviors weaken user-related behaviors, and this study enriches the boundary conditions of the role of perceived value on social attachment.

### Practical Implications

First, customer participation in the context of community group buying platform is an important source of purchase intention. In the community group buying platform, the personalized recommendation function of big data is used to regularly recommend shopping information for users’ preferences, which saves customers’ cost of searching for information, improves customers’ information sharing ability, cultivates users’ loyalty and satisfaction, and builds the brand of the community group platform, so as to provide actionable strategies for customers’ purchasing behaviors and stimulate their purchasing intentions through information sharing. The responsibility of the virtual brand community mainly refers to the clarification of the responsibilities of community members and community managers. For customer participation, it is more about community members cooperating and assisting the work of community managers, so as to make the delivery process of community services smoother. In addition, the community group purchase platform can carry out related activities through the community group purchase platform, so that the purchaser to participate in the interaction of the platform, in the interaction of the customer because of its knowledge and experience advantage, so that they feel as a member of the community group purchase platform, and enjoy the community group purchase platform “master” consciousness, so as to play its enthusiasm in the community group purchase platform, and then the community group purchase platform activities to cooperate, so that to a certain extent the customer becomes the community group purchase platform think tank, so that the community group purchase platform in product development and innovation has a strong competitive edge.

Second, customer community identification in community group buying platforms is a key factor influencing customers’ purchase intention. The research results show that the impact and results caused by customer participation behavior are not the same. One is that community group buying platform cannot underestimate the utility of customer participation behavior, and the other is that community group buying platform should not deliberately exaggerate the function of customer participation. Therefore, community group-buying platforms need to accurately identify the stimulus factors of customer participation behavior and measure customer participation behavior to produce relevant results, so as to reach a correct understanding of customer participation behavior. The community group buying platform should investigate the community identity factors caused by customer engagement behavior, specifically: First, the community group buying platform should identify the changes in customer community identity caused by customer engagement. For example, by participating in the activities of the community group-buying platform, users can achieve the same purpose as the community group-buying platform, whether they can recognize and identify with the values represented by the brand, whether they can agree and recognize a certain lifestyle represented by the brand, whether they can be convinced that the brand can bring a sense of superiority, etc. What’s more, it is necessary for community group buying platforms to clarify the changes caused by customer participation in their community identity. For example, by participating in the activities of the community group-buying platform, customers can show the change of community identity among customers brought by customer participation. For example, by participating in the activities of the community group-buying platform, users form a sense of belonging and identity to the community group-buying platform and consider themselves as important participants of the virtual community group-buying platform.

Third, the interpersonal interaction of customers in community group-buying platform is an effective way to form customers’ purchase. It is necessary for community group buying platforms to effectively guide and manage the interpersonal interactions of customers. Community group-buying platforms use a variety of methods to train existing and potential customers, help them improve their relevant knowledge and skills, and enable them to gain the strength to be able to participate in the service contact process. First of all, customer participation needs are classified, and customer needs are used as the starting point for targeted services to achieve the platform’s proposition of creating customer participation initiatives as well as shaping brand advantages by satisfying customer participation needs. Therefore, it is necessary to push the psychological changes of customers, cater to their psychological needs, and enhance the identification of customer participation. Second, it is important to create unique engagement experiences tailored to the customer’s characteristics. It is necessary for the community group buying platform to tailor the participation experience to meet the characteristics of the users themselves. At the same time, it is necessary to highlight the brand image of the community, so that customers can actively create brand associations and associate the participation experience, platform, and products to establish the community identity of the users of the virtual shopping platform. Third, the platform should achieve a highly interactive participation experience for customers. Ultimately, the platform and users, users and users can interact with each other through the community group shopping platform, and the community group shopping platform can monitor the changes in their needs and meet them in a timely manner according to the interaction between customers and get effective feedback messages to the platform, so as to maintain a long and positive interaction with customers and establish a sustainable customer communication mechanism.

### Research Limitations and Future Research Directions

In this study, the theoretical model of consumer participation influencing purchase intention is tested through SEM, and the intrinsic mechanism of consumer participation influencing purchase intention and its boundary conditions are better verified. However, as the psychological characteristics of customers’ purchase intentions, which have strong individual experiences and feelings, involved in this study, it is obviously not enough to conduct quantitative research only from a relatively static and horizontal perspective, which cannot fully penetrate the dynamic characteristics of purchase intentions itself.” Therefore, this study encourages researchers to adopt research methods that have the advantage of dynamism, such as qualitative research, in order to better capture and describe in detail the development of purchase intention in a dynamic way.

## Data Availability Statement

The raw data supporting the conclusions of this article will be made available by the authors, without undue reservation.

## Author Contributions

TH: conceptualization, methodology, and validation. JL: investigation. TH and JL: formal analysis, visualization, and writing – original draft preparation, review and editing. Both authors read and agreed to the published version of the manuscript.

## Conflict of Interest

The authors declare that the research was conducted in the absence of any commercial or financial relationships that could be construed as a potential conflict of interest.

## Publisher’s Note

All claims expressed in this article are solely those of the authors and do not necessarily represent those of their affiliated organizations, or those of the publisher, the editors and the reviewers. Any product that may be evaluated in this article, or claim that may be made by its manufacturer, is not guaranteed or endorsed by the publisher.
